# Population structure and mixed reproductive strategies in *Bipolaris maydis* from single and multiple corn cultivars in Fujian Province, China

**DOI:** 10.3389/fpls.2023.1232414

**Published:** 2023-10-03

**Authors:** Yuli Dai, Lin Gan, Chengzhong Lan, Xiaofei Liu, Wende Liu, Xiujuan Yang

**Affiliations:** ^1^ Fujian Key Laboratory for Monitoring and Integrated Management of Crop Pests, Institute of Plant Protection, Fujian Academy of Agricultural Sciences, Fuzhou, Fujian, China; ^2^ State Key Laboratory for Biology of Plant Diseases and Insect Pests, Institute of Plant Protection, Chinese Academy of Agricultural Sciences, Beijing, China

**Keywords:** *Bipolaris maydis*, reproductive strategy, genetic structure, haplotypic diversity, genetic differentiation, mating type

## Abstract

*Bipolaris maydis* is the pathogenic microorganism of southern corn leaf blight, a persistent biotic constraint responsible for substantial yield losses of corn worldwide. In the present study, 96 isolates from six representative fields growing single and multiple sweet corn cultivars in Pingnan, Fuqing, and Jian’ou in Fujian Province, which are characterized by different geographical characteristics and cropping patterns, were genetically analyzed using inter-simple sequence repeat (ISSR) markers to assess the impact of geographical origins and corn cultivars on *B. maydis* population differentiation. *B. maydis* isolates originated from diverse regions possessed higher genetic variety than those from single and multiple sweet corn cultivars. Phylogenetic analysis showed that the isolates from single and multiple sweet corn cultivars were randomly grouped into different clusters, with those from the same location tending to form clusters. A greater genetic differentiation among different geographical populations than between those from single and multiple sweet corn cultivars was observed by pairwise comparison. Hierarchical analysis indicated that among-population variation was higher when comparatively analyzed *B. maydis* populations from different locations than in those from single and multiple sweet corn cultivars. In conclusion, these results suggest that geographical origin acts a more considerable role in genetic differentiation of *B. maydis* than corn cultivar. Two divided genetic clusters were detected in the *B. maydis* populations from single and multiple sweet corn cultivars at the three locations in Fujian Province, with major genetic variation being derived within populations. The high haplotypic diversity and expected mating type ratio of 1:1 in combination with significant linkage disequilibrium suggested that a mixed reproductive strategy occurs in the *B. maydis* population in Fujian Province. This study will enrich the information on the role that geographical origins and corn cultivars play in the population structure of the pathogen as well as the reproductive strategies in *B. maydis* population in Fujian Province.

## Introduction

The foliar fungal pathogen *Bipolaris maydis* (Nisikado et Miyake) Shoem (teleomorph, *Cochliobolus heterostrophus* (Drechsler) Drechsler), the causative microorganism of southern corn leaf blight (SCLB), is one of the most important biotic restrictive factors, and contributes to substantial yield losses worldwide on corn (*Zea mays* L.), which is a crucial source of human food, industrial ingredient, and animal feed (Zwonitzer et al., 2009; Wang et al., 2014a). The SCLB symptoms initially appear on the bottom corn leaves, whereafter diffuse to the mid leaves. In susceptible corn cultivars, the necrotic lesions expand and cause significant harm to the functional leaves, thereby dramatically impairing the photosynthetic ability of corn plants (Chen and Xu, 2007). Despite the occurrence of SCLB outbreaks worldwide resulting in more than 58% yield losses in susceptible corn cultivars, these epidemics incline to be most continual in warm and moist regions (Carson et al., 2004).

There are several control strategies for SCLB in the field, such as the application of fungicides, use of resistant cultivars, and crop rotation (Wang et al., 2006). Nevertheless, despite the development of several available measures to reduce SCLB severity under natural conditions, this destructive disease still causes massive grain output losses ranging from 10% to 50% being reported in Fujian Province, China (Dai et al., 2020a). The warm and moist conditions conducive to the epidemic of SCLB are remarkably representative of Fujian, wherein the disease has developed as a crucial constraint affecting corn production (Dai et al., 2020a). Further constraints that have been responsible for enhancing the epidemics of SCLB in Fujian are the resistance development to demethylation inhibitor fungicides (Gan et al., 2016), susceptibility of sweet corn cultivars (Dai et al., 2020a), few genes that afford complete immunity to the disease (Kump et al., 2011; Li et al., 2018), and an increase in continuous corn cropping, which have facilitated massive accumulation of initial infections in the field (Dai et al., 2020a).

Information on the plant pathogens’ genetic structure is crucial to comprehend the influencing factors that contribute to their variability (McDonald, 1997; Ahmadpour et al., 2018). Additionally, this knowledge could provide suggestions for formulating available disease management strategies and guidance for further resistance breeding efforts. Molecular marker techniques have been broadly applied to analyzing genetic diversity and population structure of plant pathogens, and several molecular marker techniques have been successfully applied to evaluating the genetic diversity of *B. maydis* populations from diverse geographical origins in different countries (Nicholson et al., 1993; Karimishahri and Sharma, 2016; Wang et al., 2017; Chang et al., 2020). Among these molecular markers, inter-simple sequence repeat (ISSR) is a powerful tool to detect genotypes in heterotic groups at DNA level with little influence of environmental conditions (Kantety et al., 1995). Furthermore, ISSR can synchronously analyze plentiful fingerprint profiles and has a remarkable capacity to sensitive and repetitive identify high level of polymorphisms in large scale genome regions (Kantety et al., 1995). Hence, it is suitable for simple and fast polymorphism identify. In two *B. maydis* populations from the Hainan Province and Huang-Huai-Hai regions of China, high DNA polymorphism was measured in each sampling region using ISSR markers, with evident association between isolate groups according to their sampling locations as concluded using a clustering analysis (Chang et al., 2020). In another previous investigation, 60 race O *B. maydis* isolates with additional control race O, T, and C isolates collected from 15 corn growing locations in South China revealed high diversity and limited correlation among phylogenetic clustering, geographic location, and the virulence matrix as determined by amplified fragment length polymorphism markers (Wang et al., 2017). Genetic variation inferred through hierarchical clustering analysis of DNA fingerprinting patterns was reported in additional studies of *B. maydis* isolates from India (Gogoi et al., 2014; Karimishahri and Sharma, 2016; Manzar et al., 2022), Pakistan (Nadeem et al., 2024), and other regions (Condon et al., 2013). However, most *B. maydis* populations in these investigations originated from a very limited number of corn cultivars. There are few comparative studies on the role of the corn cultivars in *B. maydis* population structure.

The natural persistence of the foliar fungus *B. maydis* is a heterothallic ascomycete that has a specific mating type (MAT) gene locus, which has two alternative allelic forms, named MAT1-1 and MAT1-2 (Turgeon et al., 1993). Despite the sexual structures of *B. maydis* (e.g., perithecium or ascospore) having not been discovered under natural conditions, they can be easily formed using nutritive substances with suitable inductive materials and proper environmental conditions in a laboratory (Dai et al., 2020a, b). Random mating interactions among individuals influence population structure and genetic diversity of the phytopathogens through restructuring of alleles in multilocus individuals, resulting in random linking between unassociated alleles (Milgroom, 1996). Sexual recombination of new genotypes could increase multilocus haplotypic diversity and benefit plant pathogens by obtaining more adaptive characteristics, such as overcoming resistance to hosts or developing resistance to fungicides (Bi et al., 2014; Ahmadpour et al., 2018). However, there are few comprehensive studies that explore the reproductive strategies of natural *B. maydis* populations.

Due to the high frequency of SCLB outbreaks and the high proportion of epidemic individuals of *B. maydis* with highly pathogenicity to local sweet corn cultivars in Fujian Province (Dai et al., 2020a), this study used 13 ISSR markers to compare the population structure and genetic diversity of natural *B. maydis* isolates from single and multiple sweet corn cultivars with various genetic backgrounds, which afforded a comprehensive assessment of the role that geographical origins and corn cultivars play in *B. maydis* genetic differentiation. In addition, we explored the potential mode of reproduction of the natural *B. maydis* population in Fujian Province. This study will enhance the information on the role that geographical origins and corn cultivars play in the population structure of *B. maydis* as well as the reproductive strategies in *B. maydis* in Fujian Province.

## Materials and methods

### Sampling locations and isolation of *Bipolaris maydis*

Fujian is located in southeast China, with warm and wet meteorological conditions. It has three typical sweet corn-growing regions, which are characterized by diverse geographical and meteorological conditions: the east high altitude and mountainous region (single-cropping in a year), the west and central mountainous regions (double-cropping in a year), and the north and south mountainous regions (triplex-cropping in a year). The east high altitude and mountainous region has many high mountain ranges, with an average elevation being more than 800 m and large difference in temperature between day and night. This temperature condition supports for only single cropping. The west and central mountainous regions characterize by abundant light and has an annual average temperature of 17 to 20°C, which satisfy double cropping. The north and south mountainous regions have an annual mean temperature of 18 to 21°C, which allows for triplex-cropping sweet corn. These geographical and meteorological features and cropping patterns impact on the growth of sweet corn and also influence the occurrence of SCLB.

**Figure 1:**
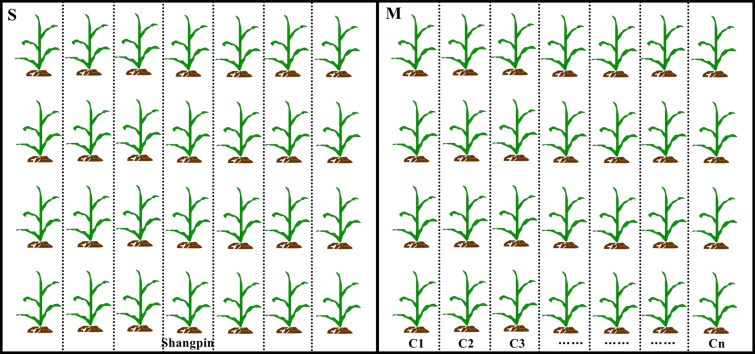
Diagram of the experimental design for corn field trials. Black boxes represent the two adjacent fields, and small plots were separated by dotted lines. S and M represent the field grown single and multiple corn cultivars, respectively. C1 to Cn represent corn cultivar 1 to cultivar n. Each cultivar was surrounded by protection plants that separated the different cultivars.

In the present study, each two adjacent fields (>600 m^2^ for each field) located in Pingnan (PN; 119°03′08″E, 27°01′31″N), Fuqing (FQ; 119°18′44″E, 25°50′10″N), and Jian’ou (JO; 118°17′08″E, 27°03′25″N), were selected for sampling collection. PN, FQ and JO situate in east, central and north Fujian, respectively. The three locations were selected for sampling due to the prolonged period of sweet corn planting and the history of frequent occurrence of SCLB. The linear distance between any two sampling sites ranges from 65 to 180 km. In each location, the both two fields were divided into 14 to 16 plots (20 m^2^ per pot; 1 m × 20 m) according to the tested number of sweet corn cultivars (**Table 1**). Each sweet corn cultivar was planted in a plot. The other entire field was planted with the SCLB-susceptible sweet corn cultivar Shangpin (Fujian Nongfeng Agriculture Development Co., Ltd., Fuzhou, China) (**Figure 1**). The same set of corn cultivars were not used in the three fields growing multiple corn cultivars in this study due to lacking of enough seeds for some cultivars. The peripheries of both fields were surrounded by an interval (1 m) with SCLB-susceptible sweet corn plants (Shangpin). The corn was cultivated following local agronomic practices. All the tested sweet corn cultivars were supplied by Fujian Seed Station. A total of 16 to 18 leaves with observable SCLB symptoms were randomly sampled from each field growing single corn cultivar, and 14 to 16 diseased leaves were randomly sampled from fields growing multiple corn cultivars (one leaf for each cultivar) when corn was approximating the anthesis stage (14-20 July, 2018). More than one isolate of *B. maydis* was randomly isolated from each leaf by surface disinfecting the lesions (5 × 5 mm) in 75% (v/v) alcohol for 2 min and in 0.1% (w/v) corrosive sublimate for 90 s, with three subsequent washes in sterile water. The sterile lesions were put onto potato dextrose agar (PDA) and cultured at 28°C under darkness for five days (Dai et al., 2020a). A small agar piece containing actively growing tip mycelia from the edge of colony was put onto a new PDA plate. Single-spore isolates were obtained according to our previously described (Dai et al., 2020a). Finally, a total of 16, 18, and 17 single-spore isolates were obtained from the single sweet corn cultivar fields in PN, FQ, and JO (labeled PN-S, FQ-S, and JO-S, respectively), and 14, 16, and 15 isolates were obtained from the multiple corn cultivar fields (labeled PN-M, FQ-M, and JO-M, respectively) (**Table 1**). The isolates of *B. maydis* were cultured on small sterile filter paper (1.0 cm × 1.5 cm) on PDA for long time storage at -20°C (Dai et al., 2020a).

**Table 1:** The number of *Bipolaris maydis* isolates and sweet corn cultivars tested in the present study, and characteristics of the three sampling locations in Fujian Province.

Sampling sites	Locations	Populations	Isolates	Corn cultivars	Year of collection	Disease severity	Characteristics of locations
Pingnan	Eastern Fujian	PN-M	PN01-M	Sukenuo 8	2018	1	Alpine (>800 m) single-cropping sweet corn regions
			PN02-M	Suyunuo 1502	2018	1	
			PN03-M	Gengyunbainuo	2018	1	
			PN04-M	Yongzhen 7	2018	1	
			PN05-M	308	2018	1	
			PN06-M	Yuetian 20	2018	3	
			PN07-M	Yuetian 22	2018	3	
			PN08-M	Rongtian 2	2018	1	
			PN09-M	Jinhuangtian 1	2018	1	
			PN10-M	Huangtian 168	2018	1	
			PN11-M	Jingui 3	2018	1	
			PN12-M	Yuetian 16	2018	3	
			PN13-M	Mintian 4	2018	1	
			PN14-M	Rongtian 1	2018	1	
		PN-S	PN15-S	Shangpin	2018	5	
			PN16-S	Shangpin	2018	5	
			PN17-S	Shangpin	2018	5	
			PN18-S	Shangpin	2018	5	
			PN19-S	Shangpin	2018	5	
			PN20-S	Shangpin	2018	5	
			PN21-S	Shangpin	2018	5	
			PN22-S	Shangpin	2018	5	
			PN23-S	Shangpin	2018	5	
			PN24-S	Shangpin	2018	5	
			PN25-S	Shangpin	2018	5	
			PN26-S	Shangpin	2018	5	
			PN27-S	Shangpin	2018	5	
			PN28-S	Shangpin	2018	5	
			PN29-S	Shangpin	2018	5	
			PN30-S	Shangpin	2018	5	
Fuqing	Central Fujian	FQ-M	FQ01-M	Jingkenuo 2000	2018	1	Mountainous double-cropping sweet corn regions
			FQ02-M	Nongkeyu 368	2018	1	
			FQ03-M	Mingyu 1203	2018	1	
			FQ04-M	Suyunuo 901	2018	1	
			FQ05-M	Meiyu 16	2018	1	
			FQ06-M	Taitian 220	2018	1	
			FQ07-M	Taitian 558	2018	1	
			FQ08-M	Taimeitian 308	2018	1	
			FQ09-M	Taimeitian 808	2018	1	
			FQ10-M	Taimeitian 809	2018	1	
			FQ11-M	Mingyu 1203	2018	1	
			FQ12-M	Shizhen	2018	1	
			FQ13-M	Min 0838	2018	1	
			FQ14-M	Min 6819	2018	1	
			FQ15-M	Min 4738	2018	1	
			FQ16-M	Min 7938	2018	1	
		FQ-S	FQ17-S	Shangpin	2018	3	
			FQ18-S	Shangpin	2018	3	
			FQ19-S	Shangpin	2018	3	
			FQ20-S	Shangpin	2018	3	
			FQ21-S	Shangpin	2018	3	
			FQ22-S	Shangpin	2018	3	
			FQ23-S	Shangpin	2018	3	
			FQ24-S	Shangpin	2018	3	
			FQ25-S	Shangpin	2018	3	
			FQ26-S	Shangpin	2018	3	
			FQ27-S	Shangpin	2018	3	
			FQ28-S	Shangpin	2018	3	
			FQ29-S	Shangpin	2018	3	
			FQ30-S	Shangpin	2018	3	
			FQ31-S	Shangpin	2018	3	
			FQ32-S	Shangpin	2018	3	
			FQ33-S	Shangpin	2018	3	
			FQ34-S	Shangpin	2018	3	
Jian’ou	Northern Fujian	JO-M	JO01-M	Rongtian 1	2018	1	Montanic triplex-cropping sweet corn regions
			JO02-M	Rongtian 2	2018	1	
			JO03-M	Mintian 6855	2018	1	
			JO04-M	Yuetian 16	2018	3	
			JO05-M	Jingui 3	2018	1	
			JO06-M	Huangtian 168	2018	1	
			JO07-M	Jinhuangtian 1	2018	1	
			JO08-M	Yuetian 22	2018	3	
			JO09-M	Yongzhen 7	2018	1	
			JO10-M	Jinguan 218	2018	1	
			JO11-M	Taitian 99	2018	1	
			JO12-M	Xianyunuo 4	2018	1	
			JO13-M	Meiyu 8	2018	1	
			JO14-M	Suyunuo 8	2018	1	
			JO15-M	Hongyu 2	2018	1	
		JO-S	JO16-S	Shangpin	2018	7	
			JO17-S	Shangpin	2018	7	
			JO18-S	Shangpin	2018	7	
			JO19-S	Shangpin	2018	7	
			JO20-S	Shangpin	2018	7	
			JO21-S	Shangpin	2018	7	
			JO22-S	Shangpin	2018	7	
			JO23-S	Shangpin	2018	7	
			JO24-S	Shangpin	2018	7	
			JO25-S	Shangpin	2018	7	
			JO26-S	Shangpin	2018	7	
			JO27-S	Shangpin	2018	7	
			JO28-S	Shangpin	2018	7	
			JO29-S	Shangpin	2018	7	
			JO30-S	Shangpin	2018	7	
			JO31-S	Shangpin	2018	7	
			JO32-S	Shangpin	2018	7	

The highest disease severity of each sampling corn cultivar was assessed using the following grading standard: 1 = less than 5% of the total leaf covering with lesions, 3 = 6 to 10% of the total leaf covering with lesions, 5 = 11 to 30% of the total leaf covering with lesions, 7 = 31 to 70% of the total leaf covering with lesions, and 9 = more than 70% of the total leaf covering with lesions (Dai et al., 2020a).

### Genomic DNA extraction

Individual isolates of *B. maydis* were grown on PDA for seven days at 28°C in the dark. Each isolate was incubated three plates. A rapid mycelial growth plate of each isolate was randomly chosen, and mycelia were lightly shaven from PDA surface in each plate and put into a sterile tube (2 mL). Genomic DNA of each isolate was extracted from mycelia using a mini CTAB extraction method (Dai et al., 2021). The concentration and quality of DNA samples were assessed using a K5800C spectrophotometer (Kaiao Technology Development Co., Ltd., Beijing, China) and diluted to 100 ng·μL^-1^ using TE buffer. The samples were stored at -20°C in a freezer for long-term storage.

### Inter-simple sequence repeat markers

Thirteen highly polymorphic ISSR-PCR primers (**Supplementary Table S1**), which were optimized from the University of British Columbia primer database (Vancouver, BC, Canada) by Dai et al. (2020a), were used for molecular markers in this study. Each 25 μL ISSR-PCR reaction was amplified in a C1000 Touch™ Thermal Cycler (Bio-Rad Laboratories, Life Science Research, Hercules, CA, USA) as described by Dai et al. (2020a). The resulting DNA fragments were isolated on 1.5% gels with 2.0 µg·mL^-1^ ethidium bromide (Shanghai Sangon Biotech Co. Ltd., Shanghai, China) and photoed using an Imaging System (Gel Doc™ XR^+^; Bio-Rad Laboratories, Life Science Research). Image Lab v5.2 (Bio-Rad Laboratories, Life Science Research) software was used for scanning DNA fingerprints on the whole piece of gel. DNA bands for each primer on gels were transformed as 0 (absence band) and 1 (presence band) for analysis.

### Genetic diversity analyses

To analyze the genetic diversity, *B. maydis* populations were divided into different groups: (1) isolates from single and multiple sweet corn cultivars in PN, FQ, and JO (all six populations: PN-S, PN-M, FQ-S, FQ-M, JO-S, and JO-M); (2) isolates from PN, FQ, and JO (three populations: PN, FQ, and JO); and (3) isolates from single (labeled S) and multiple (labeled M) sweet corn cultivars (two populations: S and M). Nei’s genetic distances among isolates or different groups were calculated using POPGENE v1.32 software according to bootstrap analysis with 1,000 replicates (Yeh et al., 1999). The percentage of polymorphic loci (*P_L_*), number of multilocus haplotypes (N_M_), number of different loci (N_L_), number of private loci (N_P_), Nei’s unbiased gene diversity (*H*_U_), and Shannon’s information indices (*I*) were inferred using GenALEx v6.5 with 999 permutations (Peakall and Smouse, 2012). Haplotypic diversity (*H_S_*) was used for indicating variety of multilocus haplotypes, which was estimated as *H_S_* = [–Σ(*P_i_* ln*P_i_*)]/ln(n), where *P_i_* represents the *i*th haplotype’s frequency and n represents population size (Groth and Roelfs, 1987). Clonal fraction (CF) was calculated according to the equation: CF = 1-[(number of unique multilocus haplotypes)/(number of isolates in the population)]. Nei’s genetic distances among individual *B. maydis* isolates and subpopulations from single and multiple sweet corn cultivars at three locations in Fujian Province were used for constructing phylograms using UPGMA method in MEGA 11 (Tamura et al., 2021).

### Genetic differentiation and population structure

To conduct genetic differentiation and population structure analyses for above-mentioned groups, phiPT values (*Φ_PT_*) with corresponding *P* values were calculated using GenALEx 6.5 (Peakall and Smouse, 2012). Randomization measurements were performed based on 999 permutations to calculate *P* values. Gene flow (Nm) was calculated as Nm = 0.5(1 – *Φ_PT_*)/*Φ_PT_* (McDermott and McDonald, 1993). Analysis of molecular variance (AMOVA) was analyzed using Nei’s genetic distance based on 999 permutations in GenAlEx 6.5 (Peakall and Smouse, 2012). Nei’s genetic distance among *B. maydis* isolates was also used for principal coordinate analysis (PCoA) using the covariance-standardized method in GenAlEx 6.5 (Peakall and Smouse, 2012). Population structure analysis was carried out in STRUCTURE v2.3.4 (Pritchard et al., 2000). Two successive independent algorithms, with burn-in period lengths of 100,000 and 100,000 repeats after burn-in, were conducted in STRUCTURE v2.3.4 software according to K (number of populations) from 1 to 10 with five replicates for each K value. The optimal K value was analyzed in accordance with the Evanno’s method (Evanno et al., 2005) by Structure Harvester (http://taylor0.biology.ucla.edu/structureHarvester/).

### Reproductive strategy in *Bipolaris maydis*

In the present study, three measures were used for investigating the potential reproduction strategy in *B. maydis* isolates from single and multiple sweet corn cultivars in the three regions in Fujian, including multilocus haplotypic diversity, the ratios of mating types, and analysis of linkage disequilibrium. The mating type of each isolate was identified by multiple PCR with two specific primer pairs (ChMAT01-3 and ChMAT02-2), which was established previously by Dai et al. (2020b). PCR amplifications were performed as described previously by Dai et al. (2020b). The resulting fragments were isolated by 1.2% gel (Biowest, Nuaillé, France) with 2.0 µg·mL^-1^ ethidium bromide (Shanghai Sangon Biotech), and the fragment sizes were estimated using a 2-kb DNA marker (Takara Biotech Co. Ltd., Dalian, China). The two mating type ratios were subjected to Pearson Chi-square tests to compare with an expected ratio of 1:1, which is expected to occur in a population characterized by sexual reproduction. Pearson Chi-square tests were performed in DPS v7.05 software (Hangzhou Reifeng Information Technology Ltd., Hangzhou, China) at a confidence interval of 95%. Two parameters of linkage disequilibrium (index of association (*I_A_*) and ${\overset{¯}{r}}_{d}$ ) were calculated using the ISSR dataset in MULTILOCUS v1.3b based on 1000 randomizations (Agapow and Burt, 2001).

## Results

### Genetic diversity analyses

In this study, a total of 167 loci were detected from 96 isolates of *B. maydis* using 13 ISSR markers, with 100% loci being polymorphic (**Supplementary Table S1**). In all six subpopulations, JO-M had the highest DNA polymorphism, with a *P_L_* of 64.7%, followed by PN-S, PN-M and JO-S with *P_L_* of 53.9%, 52.1% and 52.1%, respectively. Subpopulations FQ-M and FQ-S had the lowest DNA polymorphisms, with *P_L_* values of 49.7% and 42.5%, respectively (**Table 2**). In the three locations, JO had the highest DNA polymorphism, with a *P_L_* of 77.3%. FQ had the lowest DNA polymorphism, with *P_L_* value of 61.7% (**Table 2**). Both M and S populations had high level of DNA polymorphisms, with *P_L_* values of 86.8% and 80.8%, respectively (**Table 2**). We detected significant differences in the genetic diversity indices of *H*_U_ and *I* among the six subpopulations (**Table 2**). The genetic diversity indices among those from different locations were multiple, with the lowest and highest values being observed from FQ and JO, respectively (**Table 2**), but the genetic diversity indices for the isolates from multiple sweet corn cultivars were higher than those from single sweet corn cultivar (neglect of sampling location) (**Table 2**). We detected a total of 96 unique multilocus haplotypes, but shared multilocus haplotypes were not observed among the *B. maydis* isolates from single and multiple sweet corn cultivars at the three sampling locations in Fujian Province (**Supplementary Table S2**). Six to 8 private loci were detected in PN and JO populations, but no private loci were detected in FQ population (**Table 2**). In addition, a high level of haplotypic diversity (*H_S_* = 1.0) and a low level of clonal fraction (CF = 0) was detected in all datasets (**Table 2**).

**Table 2:** Genetic diversity of *Bipolaris maydis* populations collected from single and multiple sweet corn cultivars at three locations in Fujian Province based on an analysis of inter-simple sequence repeat markers.

Population	n	*P_L_*	N_M_	N_L_	N_P_	*Hs*	CF	*H*_U_	*I*
PN-M	14	52.1%	14	103	8	1.00	0	0.179 ± 0.015	0.255 ± 0.021
PN-S	16	53.9%	16	109	6	1.00	0	0.167 ± 0.015	0.244 ± 0.020
FQ-M	16	49.7%	16	93	0	1.00	0	0.157 ± 0.014	0.229 ± 0.020
FQ-S	18	42.5%	18	83	0	1.00	0	0.124 ± 0.013	0.185 ± 0.019
JO-M	15	64.7%	15	119	8	1.00	0	0.218 ± 0.015	0.313 ± 0.020
JO-S	17	52.1%	17	103	6	1.00	0	0.145 ± 0.014	0.218 ± 0.019
PN	30	72.5%	30	131	14	1.00	0	0.180 ± 0.013	0.282 ± 0.018
FQ	34	61.7%	34	111	0	1.00	0	0.153 ± 0.013	0.240 ± 0.018
JO	32	77.3%	32	135	14	1.00	0	0.194 ± 0.013	0.304 ± 0.018
M	45	86.8%	45	151	16	1.00	0	0.208 ± 0.012	0.331 ± 0.016
S	51	80.8%	51	141	12	1.00	0	0.174 ± 0.012	0.280 ± 0.017

PN, FQ, and JO represent *B. maydis* isolates were collected from Pingnan, Fuqing, and Jian’ou regions in Fujian Province, respectively. S and M represent isolates were collected from the single and multiple corn cultivars, respectively. n, number of isolates; *P_L_*, Percentage of polymorphic loci; N_M_, number of unique multilocus haplotypes; N_L_, number of different loci; N_P_, number of private loci; *H_S_*, haplotypic diversity, calculated as *H_S_* = [–Σ(*P_i_* ln*P_i_*)]/ln(n), where *P_i_* is the frequency of the ith haplotype in one population and n is the population size; CF, clonal fraction, calculated as CF = 1-[(number of unique multilocus haplotypes)/(number of isolates in the population)]; *H*_U_, Nei’s (1987) unbiased gene diversity; and *I*, Shannon’s information index.

**Figure 2:**
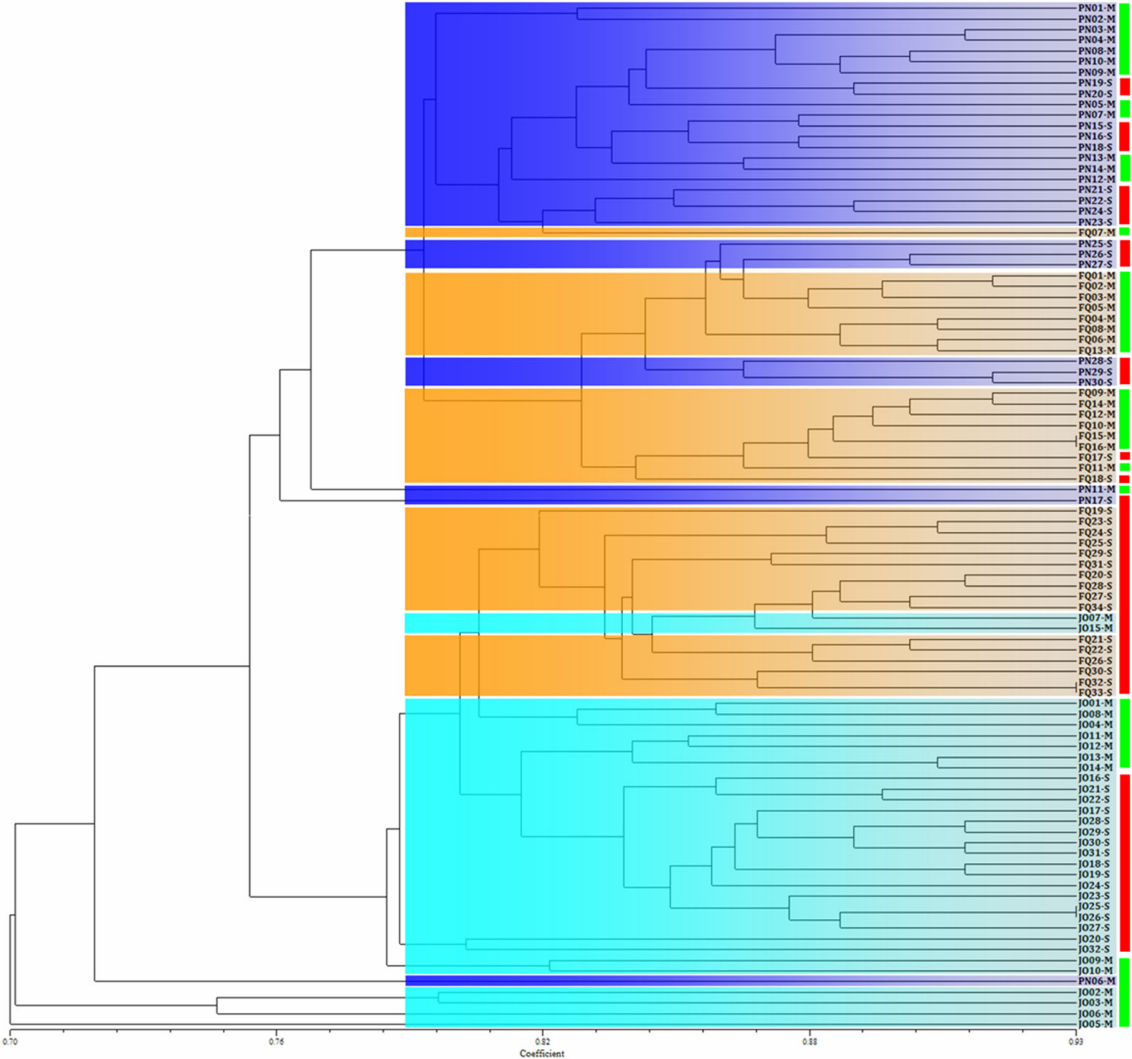
Phylogram of *Bipolaris maydis* isolates from the single and multiple corn cultivars at three sampling locations in Fujian Province based on inter-simple sequence repeat markers. Blue, orange, and cyan blocks are used to indicate *B. maydis* isolates collected from Pingnan (PN), Fuqing (FQ), and Jian’ou (JO) regions in Fujian Province, respectively. Red and green boxes on the right are used to indicate *B. maydis* isolates collected from single (S) and multiple (M) corn cultivars, respectively. *B. maydis* isolates from the single and multiple corn cultivars were randomly distributed into different clusters, whereas the isolates from the same location tended to gather together.

A phylogram of geographical *B. maydis* populations from single and multiple sweet corn cultivars was constructed according to Nei’s genetic distance (**Supplementary Figure S1**). The considerably high similarity among the *B. maydis* isolates originating from the same sampling location (even from different sweet corn cultivars) revealed that the geographical origin has a more important influence on *B. maydis* population than that of corn cultivars. Similar results were also obtained from the phylogenetic clustering of the individual *B. maydis* isolates from single and multiple sweet corn cultivars at the three sampling locations in Fujian Province, which shows that the isolates from single and multiple sweet corn cultivars were randomly grouped into different clusters, while those from the same location tended to cluster together (**Figure 2**). The results from population genetic analyses suggest that *B. maydis* isolates from different locations tended to be more different than those from single and multiple sweet corn cultivars.

### Genetic differentiation and population structure

Pairwise matrices of *Φ_PT_* and Nm values were compared to assess the genetic differentiation and gene flow between the *B. maydis* populations, and the results indicated low genetic differentiation among the subpopulations from single and multiple sweet corn cultivars within PN, with the *Φ_PT_* and Nm value of 0.082 and 5.598 (**Table 3**). However, a significant genetic differentiation was detected between the subpopulations from single and multiple sweet corn cultivars within FQ and JO, with the *Φ_PT_* and Nm values of 0.163 and 2.567, and 0.140 and 3.071, respectively (**Table 3**). Moreover, a moderate to high level of genetic differentiation (*Φ_PT_* values ranging from 0.064 to 0.283) was observed between the compared populations from single and multiple sweet corn cultivars in different locations (**Table 3**). The *Φ_PT_* values between the populations from different locations (regardless of corn cultivar) (ranging from 0.108 to 0.168) were significantly higher than those from the single and multiple sweet corn cultivars (regardless of sampling location) (*Φ_PT_* = 0.038) (**Table 3**), implying that geographical origins have a greater influence on populations than that of corn cultivars. In addition, a high level of gene flow (Nm > 4.0) was detected between the *B. maydis* populations from single and multiple sweet corn cultivars and between those from PN and FQ (**Table 3**).

**Table 3:** Pairwise matrices of genetic differentiation (*Φ_PT_*) with *P* values (above diagonal) and gene flow (Nm; below diagonal) of *Bipolaris maydis* populations obtained from single and multiple sweet corn cultivars at three locations in Fujian Province.

Population	Genetic differentiation (*Φ_PT_*)										
	PN-M	PN-S	FQ-M	FQ-S	JO-M	JO-S	PN	FQ	JO	M	S
PN-M	…	0.082^***^	0.129^***^	0.258^***^	0.188^***^	0.266^***^					
PN-S	5.598	…	0.064^***^	0.200^***^	0.150^***^	0.271^***^					
FQ-M	3.376	7.313	…	0.163^***^	0.159^***^	0.283^***^					
FQ-S	1.438	2.000	2.567	…	0.124^***^	0.224^***^					
JO-M	2.160	2.833	2.645	3.532	…	0.140^***^					
JO-S	1.380	1.345	1.267	1.732	3.071	…					
PN								0.108^***^	0.168^***^		
FQ							4.130	…	0.126^***^		
JO							2.476	3.468			
M											0.038^***^
S										12.658	
	Gene flow (Nm)										

PN, FQ, and JO represent *B. maydis* isolates were collected from Pingnan, Fuqing, and Jian’ou regions in Fujian Province, respectively. S and M represent isolates were collected from the single and multiple corn cultivars, respectively. *P* values based on 999 permutations are shown in the above diagonal, ***: *P* < 0.001. Gene flow (Nm) was estimated as Nm = 0.5(1 – *Φ_PT_)*/*Φ_PT_* (McDermott and McDonald, 1993).

The AMOVA analysis of all six populations indicated that the variation within and among populations was 82.0% and 18.0%, respectively (**Table 4**). The variations within and among populations were 87.0% and 13.0%, and 96.0% and 4.0%, when isolates were grouped based on sampling locations (regardless of corn cultivar) and corn cultivars (regardless of sampling location), respectively (**Table 4**), suggesting that the primary provenience of genetic variation in *B. maydis* populations in Fujian was originated from within populations, and much more abundant genetic diversity existed in regional populations. In addition, the *Φ_PT_* values among the three geographical populations and between those from the single and multiple sweet corn cultivars were 0.135 (*P*<0.001) and 0.038 (*P*<0.001), respectively (**Table 4**), indicating the emergence genetic differentiation among *B. maydis* populations from different locations, although not between populations from the single and multiple sweet corn cultivars. The results accordingly provide evidence that geographical origin has a remarkable impact on genetic differentiation of *B. maydis* populations in Fujian Province.

**Table 4:** Analysis of molecular variance (AMOVA) of *Bipolaris maydis* populations collected from single and multiple sweet corn cultivars at three locations in Fujian Province using inter-simple sequence repeat data.

Source	Degree of freedom	Sum of squares	Mean of squares	Estimated variance	Percentage of total variance	*Φ_PT_*	*P* value
Among populations from single and multiple corn cultivars at the three locations							
Among Pops	5	312.670	62.534	3.062	18.0%	0.184	<0.001
Within Pops	90	1224.517	13.606	13.606	82.0%	…	…
Total	95	1537.188		16.668	100%	…	…
Among populations from different locations (neglect of corn cultivars)							
Among Pops	2	175.225	87.613	2.283	13.0%	0.135	<0.001
Within Pops	93	1361.962	14.645	14.645	87.0%	…	…
Total	95	1537.188		16.928	100%	…	…
Between populations from single and multiple corn cultivars (neglect of sampling location)							
Among Pops	1	45.705	45.705	0.624	4.0%	0.038	<0.001
Within Pops	94	1491.482	15.867	15.867	96.0%	…	…
Total	95	1537.188		16.491	100%	…	…

**Figure 3:**
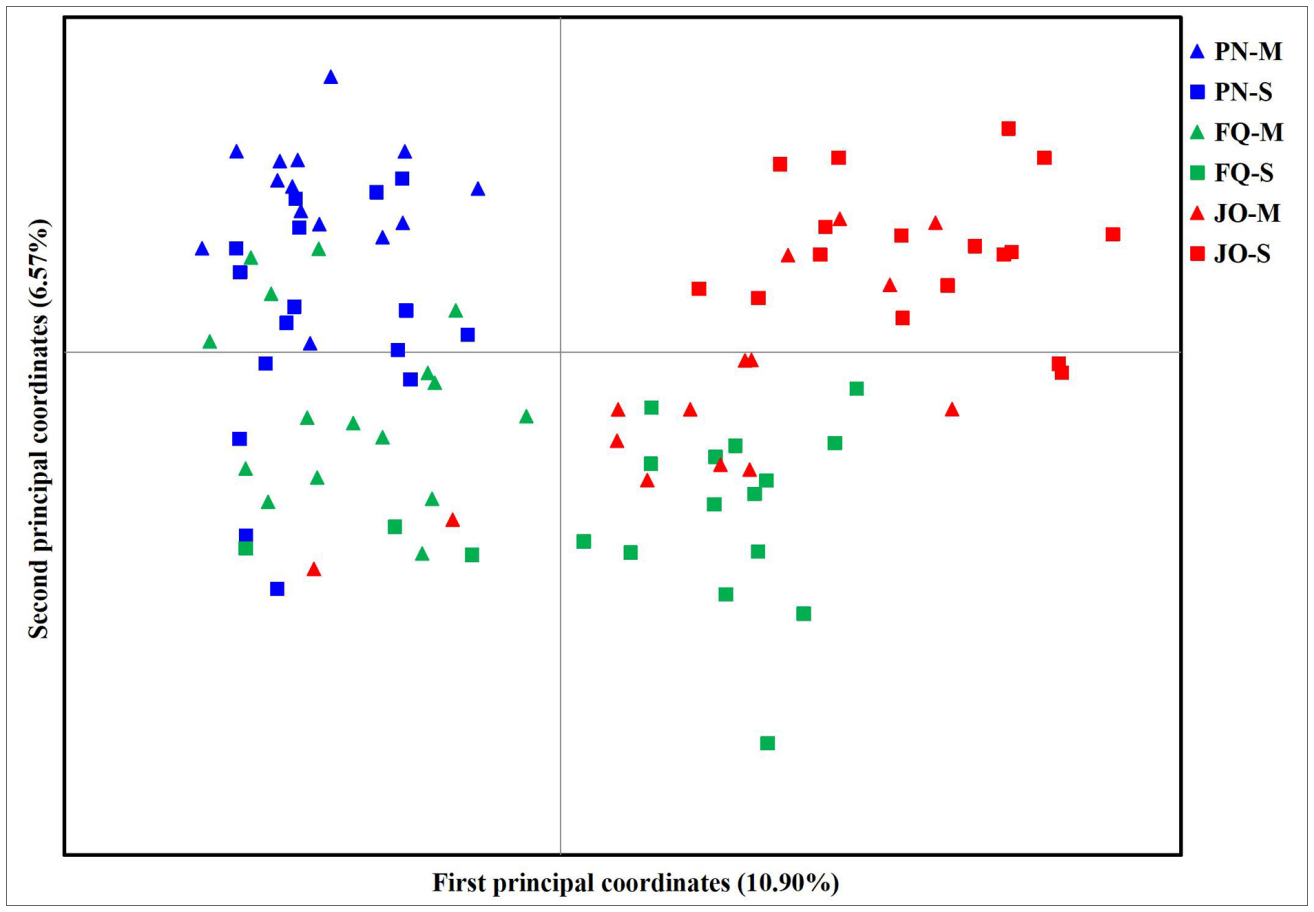
Principal component analysis (PCoA) based on 13 ISSR markers for 96 individual isolates from the single and multiple sweet corn cultivars at three locations in Fujian Province. PN, FQ, and JO represent *B. maydis* isolates were collected from Pingnan, Fuqing, and Jian’ou regions in Fujian Province, respectively. S and M represent isolates were collected from the single and multiple corn cultivars, respectively. Individual isolates from the single and multiple sweet corn cultivars at the same location are marked using the same color with different symbols. The first and second principal coordinates account for 10.90% and 6.57% of the variation, respectively.

The PCoA results showed two separate groups, with all isolates from PN (including PN-S and PN-M) and numerous isolates from FQ-M at the left of the vertical axis and the majority of isolates from JO (JO-S and JO-M) and FQ-S at the right of the vertical axis (**Figure 3**). The first and second principal coordinates accounted for only 17.47% of the total variation, with isolates from the same location tending to group together (with the exception of FQ) (**Figure 3**), suggesting no obvious separation among *B. maydis* isolates from single and multiple sweet corn cultivars, but a tendency of deviation among those from different sampling locations. Therefore, collectively, the results from the population genetic analyses revealed that the geographical origin plays a more crucial role than that of corn cultivars in *B. maydis* population differentiation in Fujian Province.

**Figure 4:**
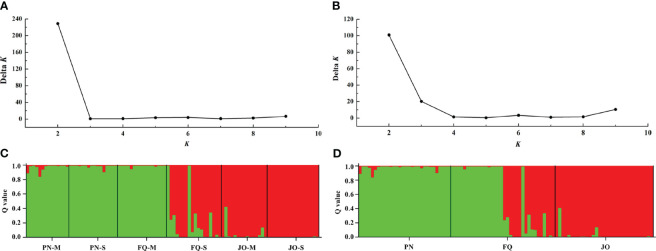
Population genetic structure-inferred membership parameter for *Bipolaris maydis* populations from the single and multiple sweet corn cultivars at three locations in Fujian Province. **(A, B)** The values of *Δk* and optimal numbers of *K* = 2 clusters. **(C, D)** The membership of each isolate for the two clusters. Each isolate is figured by a thin perpendicular bar and the length of each colored bar represents the membership parameter for each cluster. Individual isolates are clustered on the basis of geographical origin and corn cultivar. Black thick lines divide individual isolates into different subpopulations. PN, FQ, and JO represent *B. maydis* isolates were collected from Pingnan, Fuqing, and Jian’ou regions in Fujian Province, respectively. S and M represent isolates were collected from the single and multiple corn cultivars, respectively.

STRUCTURE analysis indicated that the optimal *K* (number of populations; highest peak of *ΔK* appearance) for all six populations and populations from sampling location were *K* = 2 (**Figures 4A, B**). The membership scores of the most *B. maydis* isolates for each population were high, but a limited number of isolates exhibited admixture (**Figures 4C, D**). The *B. maydis* isolates from PN and FQ-M were divided into one of two genetic clusters, and the isolates from JO and FQ-S were divided into another cluster, indicating a tendency of association between genetic clusters and geographical populations. Thus, the STRUCTURE analysis combined with those from the PCoA supported that *B. maydis* isolates from single and multiple sweet corn cultivars at the three locations in Fujian divided into two different genetic populations.

### Reproductive strategy in *Bipolaris maydis*

High multilocus haplotypic diversity, an expected ratio of 1:1 between the two mating types, and linkage equilibrium would be indicative of the potential sexual reproduction in a fungal population. In the present study, the multilocus haplotypic diversity values were considerably high (*Hs* = 1.0) in all datasets (**Table 2**). Near-equal ratios were also detected between MAT1-1 and MAT1-2 from single and multiple sweet corn cultivars at the three sampling locations (**Table 5**), suggesting the potential sexual reproduction in the *B. maydis* populations from single and multiple sweet corn cultivars in Fujian Province. The detected indices of linkage disequilibrium (*I*_A_ and *r̄_d_*) for the *B. maydis* populations from single and multiple sweet corn cultivars in Fujian Province were significantly different in all the datasets, thus rejecting the hypothesis of random mating (**Table 5**).

**Table 5:** Multilocus linkage disequilibrium analysis of *Bipolaris maydis* populations collected from single and multiple sweet corn cultivars in Fujian Province.

Population^a^	Year of collection	Mating types		Linkage disequilibrium analysis ^c^		
		MAT1-1:MAT1-2	*P* value ^b^	*I*_A_	${\overset{¯}{r}}_{d}$	*P* value (*I*_A_ & ${\overset{¯}{r}}_{d}$ )
Among populations from single and multiple corn cultivars at the three locations						
PN-M	2018	3:11	0.061	1.26	0.013	< 0.001
PN-S	2018	8:8	0.803	1.23	0.013	< 0.001
FQ-M	2018	8:8	0.803	1.04	0.014	0.001
FQ-S	2018	7:11	0.480	2.07	0.023	< 0.001
JO-M	2018	8:7	1.000	2.59	0.024	< 0.001
JO-S	2018	9:8	1.000	0.83	0.010	< 0.001
Among populations from different locations (neglect of corn cultivars)						
PN	2018	11:19	0.201	0.85	0.007	< 0.01
FQ	2018	15:19	0.607	1.74	0.016	< 0.01
JO	2018	17:15	0.860	3.06	0.024	< 0.01
Between populations from single and multiple corn cultivars (neglect of sampling location)						
M	2018	19:26	0.371	2.75	0.020	< 0.01
S	2018	24:27	0.779	1.82	0.013	< 0.01
Total		43:53	0.358	1.79	0.012	< 0.01

a PN, FQ, and JO represent *B. maydis* isolates were collected from Pingnan, Fuqing, and Jian’ou regions in Fujian Province, respectively. S and M represent isolates were collected from the single and multiple corn cultivars, respectively.

b *P* values were calculated by analysis of mating type ratios using Pearson Chi-square tests in DPS software v7.05 (Hangzhou Reifeng Information Technology Ltd., Hangzhou, China). *P*<0.05 indicates a skewed 1:1 mating type ratio and rejects randomly mating within population.

c Values of *I*_A_ (index of association) and ${\overset{¯}{r}}_{d}$ (multilocus linkage disequilibrium) were calculated by analysis of the ISSR markers data with 1000 randomizations using MULTILOCUS v1.3b (Agapow and Burt, 2001). ${\overset{¯}{r}}_{d}$ values that deviate remarkably (*P*<0.05) from 0 indicate an obvious linkage disequilibrium and support clonal reproducing.

## Discussion

In the present study, population genetic analyses were used to assess the major role of geographical origins and corn cultivars in *B. maydis* population differentiation in Fujian Province. The results indicated that significant differences in genetic diversity were observed in the *B. maydis* populations from different geographical locations, with high deviations in genetic diversity being observed in those from single and multiple sweet corn cultivars, implying that Fujian *B. maydis* isolates showed a greater genetic association with geographic origin and corn cultivars. A moderate to high genetic differentiation (*Φ_PT_* > 0.1, *P*<0.001) was detected between the *B. maydis* isolates from the three sampling locations, which was higher than that when isolates were grouped by corn cultivar (*Φ_PT_* <0.04, *P*<0.001). In addition, the genetic variation among populations was higher when isolates were grouped based on location (13.0%), compared to that when isolates were grouped according to corn cultivar (4.0%). The results obtained in the present study show that low genetic similarity and higher genetic differentiation being detected among *B. maydis* isolates from different sampling locations but not different corn cultivars implies that the geographical factor played a more important role in the population differentiation of *B. maydis* isolates than corn cultivars. The main reason for lack of genetic differentiation in *B. maydis* populations from single and multiple sweet corn cultivars may be due to high level of gene exchange (Nm > 12) within population. The findings in this study were consistent with analogical investigations revealing genetic differentiation of *B. maydis* on corn from north and south China and *Villosiclava virens* on rice in the Hubei Province of China, where both geographical origins and cultivars had an impact on the differentiation of *B. maydis* and *V. virens* populations, but the geographical origins played a more important role in the selection of *B. maydis* and *V. virens* isolates when compared with that of cultivars (Wang et al., 2014b; Chang et al., 2020). However, the AMOVA results from our study were in contrast with previously similar studies exhibiting host differences of *Exserohilum turcicum* and *Monolinia fructicola* on different cereal crops (corn and sorghum) and fruits (apricot, cherry, peach, and plum), respectively, where among-population differentiation was higher when isolates were grouped based on host rather than geographical location (21% and 2%, *P* < 0.022; 24% and 7%, *P* < 0.001, respectively) (Papavasileiou et al., 2015; Nieuwoudt et al., 2018).

Plant pathogen populations with few private loci are usually characterized by a lack of population subdivision and continual gene drift among isolates from different regions (Slatkin, 1995). In the present study, a limited number of private loci were observed among the *B. maydis* populations from single and diverse sweet corn cultivars at three sampling locations in the Fujian Province, which was consistent with the findings reported by Onaga et al. (2015), who detected few private loci in populations of another important pathogen, *Magnaporthe oryzae*, from East Africa with high gene flow between subpopulations. Nevertheless, our results are in contrast with those from a similar population genetic study of *E. turcicum* in South Africa, where numerous private alleles were observed in *E. turcicum* populations from corn and sorghum with limited gene flow between the two hosts (Nieuwoudt et al., 2018). The limited number of private loci and absence of shared multilocus haplotypes between isolates from the single and multiple corn cultivars imply that high levels of gene flow resulted in no genetic differentiation between the *B. maydis* isolates from single and multiple sweet corn cultivars in Fujian Province. This inference was supported by the results from our population structure and genetic differentiation analyses, in which a high level of gene flow (Nm > 12) was detected between the populations from the single and multiple corn cultivars in Fujian Province. In addition, high levels of haplotypic diversity (*H_S_* =1.0) were detected from *B. maydis* populations in all situations, suggesting that multiple infection sources occur on individual sampling fields. Meanwhile, no shared multilocus haplotypes were detected from the single and multiple corn cultivars at the three locations in Fujian Province, indicating that large scale migration of *B. maydis* isolates did not occur in Fujian Province. Furthermore, disease severities in the three sampling fields growing multiple sweet corn cultivars were lower than that growing single sweet corn cultivar (**Table 1**), suggesting rational distribution of resistant and susceptible corn cultivars being used for effective control SCLB.

Heterothallic plant pathogens undergoing frequent sexual reproduction are commonly characterized by high haplotypic diversity, equal mating type distribution, and linkage equilibrium (Milgroom, 1996). In the present study, we used these three approaches to evaluate the potential reproductive strategies of *B. maydis* populations from single and multiple sweet corn cultivars at three locations in Fujian Province. The detected haplotypic diversity values were relatively high in the *B. maydis* populations (1.00) from single and multiple sweet corn cultivars in Fujian Province. These high levels of haplotypic diversity were not related to a strictly clonal population, which afford proof for sexual reproduction. The haplotypic diversity values of the *B. maydis* isolates were consistent with those of high-haplotypic diversity populations of *Cercospora sojina* (0.41–0.69) (Kim et al., 2013), *Mycosphaerella musicola* (0.55–0.89) (Hayden et al., 2005), *Bipolaris oryzae* (0.51–1.00) (Castell-Miller and Samac, 2012), *Setosphaeria turcica* (0.81–0.91) (Ferguson and Carson, 2007), and *Dothistroma septosporum* (0.94–1.00) (Dale et al., 2011) which were reported in similar population genetic studies characterized by frequent sexual reproduction. The values obtained for the two mating type ratios of the *B. maydis* isolates from all three datasets showed no significant difference from the expected 1:1 ratio using the full ISSR datasets, confirming the hypothesis of frequency-dependent selection (Milgroom, 1996; May et al., 1999) and providing evidence of random mating interactions. However, significant differences in linkage disequilibrium (*I_A_* and *r̄_d_*) (*P* < 0.01) were detected between populations in all datasets; therefore, we gained no forceful proof in favor of random mating in the Fujian *B. maydis* populations. Previous studies suggested that many factors (e.g. gene drift and flow, selection) could result in linkage disequilibrium analysis deviation from random mating, despite linkage disequilibrium is a crucial measurement for random mating (Milgroom, 1996; Mullett et al., 2015). As the gene drift between some sampling locations is high, especially the locations cultivated the same crop, gene flow may well impact on linkage disequilibrium at these locations. In addition, the gene exchanges between sampling locations and between STRUCTURE population groups was detected to be high, hence high the effect of gene flow on linkage disequilibrium in these populations (Milgroom, 1996). This is likely a contributing factor in this study.

Contradicting results were obtained from the analyses of haplotypic diversity, ratios of the two mating types, and linkage disequilibrium. The high levels of haplotypic diversity and equal ratios of mating type supported that sexual reproduction is considerable in the *B. maydis* lifecycle in Fujian Province despite evident proof of linkage disequilibrium. In similar previous investigations of other *Cercospora*, *Dothistroma*, and *Exserohilum* fungi, although linkage disequilibrium analysis did not support sexual reproduction in all datasets tested, high haplotypic diversities and near equal mating type ratios suggested that these populations experience both sexual and frequent clonal reproduction (Dale et al., 2011; Kim et al., 2013; Human et al., 2016; Nieuwoudt et al., 2018). Thus, the conflicting results between the haplotypic diversity, mating type distribution, and linkage disequilibrium analyses obtained in the present study imply a mixed reproductive strategy within the *B. maydis* populations from single and multiple sweet corn cultivars in Fujian Province. This reproductive strategy was beneficial to *B. maydis* adaptability. Because wide new genotypes generating in sexual reproduction could help the pathogen to obtain more adaptive characteristics, such as overcoming host resistance or developing fungicides resistance (McDonald and Linde, 2002; Bi et al., 2014). The perithecium produced in sexual reproduction is a good protective structure to help *B. maydis* survival in the adverse environment. In addition, asexual reproduction is beneficial to rapid growth and expansion. This challenge warrants the utilization of management strategies to prolong the service life period of these control methods (Liu and Mundt, 2020). Furthermore, the results in this study also showed that neither geographical origins nor corn cultivars have an impact on reproductive strategy of *B. maydis* populations. The main reason may be that the reproductive strategy of *B. maydis* is not only influenced by environmental factors, but by other factors, such as mating type distribution, alternative hosts.

Collectively, the results of STRUCTURE analysis showed that two genetic clusters occur in the *B. maydis* population from single and multiple sweet corn cultivars in Fujian Province. Intriguingly, *B. maydis* populations from single and multiple sweet corn cultivars at FQ exhibited obvious cultivar-specific differences, although the isolates of *B. maydis* were collected from the two neighboring fields. A reasonable interpretation of this situation is that there are two different proveniences of the pathogen that undergo mixing through sexual reproduction. A mixed reproduction strategy might explain the consolidation of two genetic groups at a comparatively slow speed (Liu and Mundt, 2020). The mechanisms of this inherent differentiation should be further considered, and continued genetic study of additional more samples, since such study is necessary, and will be beneficial to clarifying this controversy.

## Conclusion

In conclusion, we comparatively studied the influence of geographical origin and corn cultivars on the differentiation of *B. maydis* populations in Fujian Province, given the more important role of the geographical origin in this pathogen differentiation. Our results showed high haplotypic diversity, an equal ratio of mating type, and remarkable linkage disequilibrium, and therefore are indicative of a mixed reproductive strategy among the *B. maydis* isolates from single and multiple sweet corn cultivars in Fujian Province. Sexual reproduction mostly took place on senescent phytodetritus at the later stage of crops or alternative hosts. Therefore, the existence of perithecia or ascospores on corn debris or alternative hosts needs to be further investigated to clarify the full lifecycle of *B. maydis*. The wide variation of gene recombination in sexual reproduction can increase the adaptability of offspring to natural selection and also promote the spread of advantaged mutations in the population. However, asexual reproduction can enable offspring to maintain the parental excellent characteristics, which can accelerate growth and achieve rapid population expansion. Hence, further evaluation of *B. maydis* is necessary to carefully assess the relative importance of both sexual and asexual stages in the lifecycle of *B. maydis*. In addition, a further temporal investigation of *B. maydis* isolates collected from the same site over continuous years will be beneficial to clarifying the influence of sampling time on genetic diversity and population structure.

## Data availability statement

The original contributions presented in the study are included in the article/**Supplementary Material**. Further inquiries can be directed to the corresponding authors.

## Author contributions

YD, XY, and WL: design the experiment. YD, LG, and CL: methodology and technology. YD, LG, CL, XL, and XY: samples and isolates collection. YD, LG, and CL: investigation and data analysis. YD: writing the draft manuscript. YD and WL: further review and editing. WL and XY: project supporting. All authors contributed to the article and approved the submitted version.
