# Supplementary material for: Population structure and mixed reproductive strategies in Bipolaris maydis from single and multiple corn cultivars in Fujian Province, China
Source: Front Plant Sci. 2023 Oct 3;14:1232414. doi: 10.3389/fpls.2023.1232414 (PMC10583543; doi:10.3389/fpls.2023.1232414)
Supplement: Supplementary file 1 [file DataSheet_1.pdf]

Supplementary Information

Supplementary Table S1. Primers used for inter-simple sequence repeat markers to evaluate the genetic diversity and population structure of *Bipolaris maydis* in the present study.

Primer codes	Primer sequences (5′→3′)	Annealing temperature (°C)	N	NPL
UBC808	AGAGAGAGAGAGAGAGC	52	12	12
UBC835	AGAGAGAGAGAGAGAGYC	55	15	15
UBC841	GAGAGAGAGAGAGAGAYC	52	14	14
UBC856	ACACACACACACACACYA	50	13	13
UBC857	ACACACACACACACACYG	53	16	16
UBC859	TGTGTGTGTGTGTGTGRC	51	18	18
UBC884	HBHAGAGAGAGAGAGAG	53	7	7
UBC888	BDBCACACACACACACA	53	9	9
UBC889	DBDACACACACACACAC	51	15	15
UBC891	HVHTGTGTGTGTGTGTG	50	13	13
DC02	GAGAGAGAGAGAGAGAHC	53	14	14
DC03	ACACACACACACACACYC	51	13	13
DC05	BVBCACACACACACACA	57	8	8
Total	…	…	167	167

Y: (C+T); R: (A+G); H: (A+T+C); B: (T+C+G); D: (A+T+G); V: (A+C+G); N: number of loci; NPL: number of polymorphic loci.

Supplementary Table S2. Sorted multilocus haplotypes of *Bipolaris maydis* from single and diverse sweet corn cultivars in Fujian obtained using 13 inter-simple sequence repeat markers in the present study.

Sample	Pop	Genotype	No.	Label
JO24-S	JO-S	0000000000000000000000000000000001111000011000011000000000011111100111100000000000000000111100000000000000000000000000000110000001100000000110000000011111100110000000000111g	1	1
JO19-S	JO-S	0000000000000000000000111100000000110000110000110000000000000001100111100000000000000000111100000000000000000000000000000011111000000000000000011000000111111001100000000111g	1	2
JO18-S	JO-S	0000000000000000000000111100000000110000110000110000000000000001111111000000110000000011110000000011000000000000000000000000000000000000000000000001111000011000000000111g	1	3
PN15-S	PN-S	0000000000000000000000111100000000110000110000110011110000000000111111000000110000000011110000000000110000000000000000000000000110000001100000000111110000110000110000001g	1	4
FQ08-M	FQ-M	0000000000000000000000111100000011110000000000100000000000000001111110000001100000000111100000000000000000000000000000000000001100000000000000000110000000000001100000g	1	5
FQ29-S	FQ-S	0000000000000000000000111100000011110000110000110000000000000001111111000000000000000000111100000000000000000000000110000110000001100000000000000000011001100000000011100111g	1	6
JO10-M	JO-M	0000000000000000000000111100000011110000110000110011000000000000011001100000000000000000111100000000011111100001100000000000000000000000001100111100111100110011000000000111g	1	7
JO01-M	JO-M	0000000000000000000011001100000011110000110000110000000000000001111111100001111110000001111000000000011000000001100000000000000000000000000000000110011001100001100001g	1	8
PN07-M	PN-M	0000000000000000000011001100000011110000110000110011000000110000111111000000110000110011110000000000000000000000000000000000001100000011000000000111100001100001100001g	1	9
FQ19-S	FQ-S	0000000000000000000011111100000011110000110000110000000000000001111111100000000000000000111100000000000011001100001100000011110011000000000000000000111111000000001100000g	1	10
JO09-M	JO-M	0000000000000000000011111100000011110000110000110000001111001111111111100001100110000000111100000000000011000000000000000000001100000000000011001111111100110000000001g	1	11
FQ13-M	FQ-M	0000000000000000011001111000000111100001100001100110000110011001111111000000110000000011110000000000000000000000000000000000001100000000000000000110000001100001100111g	1	12
FQ06-M	FQ-M	0000000000000000011001111100111111000011000011000000001100000011001100001111000000000111100000000000001111000000000000000000001100000000000000000110000001100001100001g	1	13
PN27-S	PN-S	0000000000001100000000000011000000111111110000110011000000000000111111000000110000110011110011000000000000000000000000000000001100000000000000000110000001100001100111g	1	14
PN18-S	PN-S	0000000000001100000000000110000000011000011000011000000000000000111111110000000000000001100000000000000000000000000000000000001100000000000000001111000000000000001100001g	1	15
JO16-S	JO-S	0000000000001100000000000110000001111000011000011000000000000111111111100000000000000000111100000000000001100000000000000111100001100000000000011000011111100000000000000001g	1	16
JO04-M	JO-M	0000000000001100000000111100000001111000011000011000000000000001111001100000000000000000110000000000000000000000000000000000001100000011000011110011001100110000110001g	1	17
JO17-S	JO-S	0000000000001100000011111100000011110011110000110000000000000111111111000000110000000011110000000011001100000011000011111111001100000000001100000011111100110000000000111g	1	18
FQ15-M	FQ-M	0000000000001100001100111100000011110000110000110011000011000000111111000000000000000001111000000110000110000000000110000000000110000000000110000001100000011000011100111g	1	19
JO21-S	JO-S	0000000000001111000011001100000011110000110000110011000000000011111111000000000000000000111100001100000000000000000000000110011001100000000000110000111111001100000000111g	1	20
JO23-S	JO-S	0000000000111100000001111110000000111100001100001100000011111100111111110000000000000000111100000000000000000000000000000011001100110000000000110000111111100110000000000111g	1	21
JO15-M	JO-M	0000001100000000000000001100000011110000110000110000000000000001111111100000000000000000111100000011000000000011110000000000000000000000000000110000001111110011000000000001g	1	22
PN25-S	PN-S	0000001100000000000000001111111111110000110000110000000000000000111111000000110000000011110000000000110011000000000000000000000110000000000000000001100001111000011100111g	1	23
FQ09-M	FQ-M	0000001100000000000000011110000000111100001100001100000000000011001111111000000000000000001111000000000110000110000000000000000000000000000000000000000000000000000110000110011 1g	1	24

FQ28-S	FQ-S	00000011000000000000011111100000011110000110000110000000000000011111111000000000000000011110000000000000000000000000000000000000000000000000000000001100110011001100001100001g	1	25
FQ14-M	FQ-M	00000011000000000000011111100000011110000110000110000000011001100111111000000000000011001111000000000000000000000000000000000000000011000000000011000000000000001100001100111g	1	26
JO20-S	JO-S	00000011000000000000011111100000011110000110000110011000011000011111111000000000000000001111111111000000110000000000000000000000011000000000000000000111111001111000000111g	1	27
PN01-M	PN-M	00000011000000000001100111111000011110000111111111111000000000000011111100000011000011001100000000000001100000000000000000000000000000000000000000000000111100001100001111111g	1	28
FQ02-M	FQ-M	00000011000000000001111001111000011110000110000111111000000000000011111100000000000000000111100000000000000000000000000000000000000011000000000000000000110000111100001100111g	1	29
PN10-M	PN-M	00000011000000000111111001111000011110000110000111100000011110000111111000000000000000001111000000000000000000000000000000000000000011000000000000000000111100001100001100001g	1	30
JO31-S	JO-S	000000110000000110000000011000000111100001100001100000000000000011111111000000110000000011110000000000000000000000000000000000000000110000000000000000001111110000000000000111g	1	31
JO32-S	JO-S	0000001100000001100000001111000000011110000110000110000000000000001111111100000000000000001111000011111100000000000000000000000000000110000000000000000001100000011000000000111g	1	32
PN08-M	PN-M	00000011000000011000011111100000011110000110000110000000011000000111111000000011001100001111000000000000000000000000000000000000000011000000000000000000111100001100001100001g	1	33
FQ11-M	FQ-M	00000011000000011001111111100000011110000110000110000000000000000011000000000000000000001100000000110000110000000011111100000000011000000000000110000110000001100001100111g	1	34
PN21-S	PN-S	00000011000000011001111111100000011110011111100110011000011001100110011000000000000000011111100000000000000000000000000000000110000110000001100000000111111000000000001100001g	1	35
FQ03-M	FQ-M	00000011000011000000000001111000000011000011000011111100000000000000111111000011110011110011110000000000000000000000001100000000000011000000000011000000110000001100001100001g	1	36
FQ31-S	FQ-S	00000011000011000000000111100000000011000011000011000000000000000111111110000000000000000111100000000000000000000011001100000000000000000000000000000000001100110000000001100111g	1	37
FQ32-S	FQ-S	00000011000011000000000111100000001111000011000011000000000000000111111110000000000000000111100000000000000000000000000000000000000011000000000000000000110011001100001100111g	1	38
FQ27-S	FQ-S	0000001100001100000000011110000000111100001100001100000000000000011111111000000000000000011110000000000011000000001111000000000000000000000000000000000110011001100001100001g	1	39
FQ33-S	FQ-S	0000001100001100000000011110000000111100001100001100000000000000011111111000000000000000011110000000110011110000000001100000000000000000000000000000000110011001100001100111g	1	40
FQ30-S	FQ-S	0000001100001100000000011110000000111100001100001100000000110011111100110000000000000000011110000000000011000000000011000000110000000000000000000000000110011001100001100111g	1	41
JO11-M	JO-M	00000011000011000000000111100000001111000011000011001100000000000111111110000000011000000011110000000001111110000000000000000000000011000000000011000000000000001100000000001g	1	42
PN23-S	PN-S	000000110000110000000001111000000011110011110000110011000011000000111111000000000000000011111100000000000011111111000000000000000000011000000000000000000110000001111001100001g	1	43
JO25-S	JO-S	000000110000110000000001111110000111100001100001100000000001100111111111000000000000000001111000000000110000000000000000000000011001100000000000000000001111110011000000000111g	1	44
PN14-M	PN-M	000000110000110000000001111110000111100001100001100110000110011111111111100000000000000001111000000000000000000000000110000000000000110000000000000000001111000000000000000000g	1	45
FQ05-M	FQ-M	000000110000110000000110000000000011110000000000011111100000000000001111110000000110000110011110000000000000111100000000011000000000011000000000000000000110000001100001100111g	1	46
JO03-M	JO-M	0000001100001100000001100000000000111100110000000110011000000000001111000000000001100000000110000000000000011110011000000000000000011111111111100000011000000000001100001100001g	1	47
FQ34-S	FQ-S	0000001100001100000001100110000000111100001100001100000000000000011111111000000110000000001111000000000000000000000000000000000000000000000000000000000001100110000000001100001g	1	48
FQ22-S	FQ-S	000000110000110000000111111000000000110000110000110000000000000000011110000000000000000000111100000000000110000110000000000000000000110000000000000000001100110000000001100111g	1	49
FQ07-M	FQ-M	000000110000110000000111111000000000110000110000110000000011001100111111000000000000001111111100000000000000000000000000000000000000011000000000000000000110000001100001100111g	1	50
FQ25-S	FQ-S	00000011000011000000011111100000011110000110000110000000000000000011111100000000000000000011110000000000000000001100000000110000110000000000000000001100110011001100001100111g	1	51
FQ21-S	FQ-S	000000110000110000000111111000000111100001100001100000000000000000111111000000000000000000111100000000000000000011000011000000000000011000000000000000000110011001100001100111g	1	52
FQ20-S	FQ-S	00000011000011000000011111100000011110000110000110000000000000000011111100000000000000000011110000000000000110011001100000000000000000000000000000000000001100000000000001100000g	1	53

FQ24-S	FQ-S	0000001100001100000011111100000011110000110000110000000000000000111111000000000000000000111100000000000111111110000110011111111001100000000000000110011001100110000110011 1g	1	54
FQ23-S	FQ-S	000000110000110000001111110000001111000011000011000000000000000111111110000000000000000011110000000000000111100000000000001100110011000000000000000110011001100110000110011 1g	1	55
FQ26-S	FQ-S	000000110000110000001111110000001111000011000011000000000110011111111110000000000000000000000000000000000000000000000000000000001100000000000000000011001100110000110011 1g	1	56
FQ17-S	FQ-S	000000110000110000001111110000001111000011000011001100001100111111100110000000000000000011110000000000000000110000000001111000000000110000000000000000000000000000011000011001 11g	1	57
FQ04-M	FQ-M	0000001100001100000011111100001111110000110000110000000001100000011111100000001100001100111100000000000000000000000000110000000000000110000000000000000001100000000000001100001g	1	58
PN16-S	PN-S	000000110000110000001111111100000011000011000011001100000000000001111111100000000000000011110000000000000000000000000000000000000000110000000000000000111100000000111100110000 1g	1	59
PN17-S	PN-S	00000011000011000000111111110000111100001100001100000000011000000111111110000110000000011110000000000000110000000011000000000000011000000000000000000011000000111100111100 1g	1	60
PN30-S	PN-S	00000011000011000011000011110000111100001100001100000000011001111111111000000000000000011110000000000000111100000000000000000000011000000000011000000110000001100001100111g	1	61
PN28-S	PN-S	00000011000011000011000011110000111100001100001100110000110011111111110000000110000110011110000000000011110000000000000000000000011000000000000000000011110000110000110011 1g	1	62
FQ18-S	FQ-S	00000011000011000011001111000000000110000110000110000000001100000011000000000000000000000111100000000000011110000000001100001100001100000000000000000001100000000000001100000g	1	63
PN24-S	PN-S	00000011000011000011001111110000111100001100001100110000110011111111110000000110000001111110000000000000000000000000000000000000011000000000000000000011000000110000110000 1g	1	64
FQ01-M	FQ-M	00000011000011000011110011000000111100001100001111110000000000000111111000011110000000011110000000000000111100000000011000011000011000000000000011000011000011110000110011 1g	1	65
PN22-S	PN-S	00000011000011000011111111000000111100001100001100000000011000000110011000000000000000011111100000000000001111110011000000000110000110000001100000000111100000001100001100001g	1	66
FQ12-M	FQ-M	00000011000011000011111111000000111100001100001100110000000011001111111000000000000000011110000000000011000000000000011110011000011000000000001111000011000000110000110011 1g	1	67
PN13-M	PN-M	00000011000011000011111111110000111100001100001100110000110011111111110000000000000000011110000000000011110000000011000000000000011000000000000000000011110000110000110000 0g	1	68
PN20-S	PN-S	00000011000011000011111111110000111100111111001100110000110011111111110000000000000000011110000000000000000000000000000000000000011000000000000000001111000000011000011001 11g	1	69
JO29-S	JO-S	00000011000011110000000001100000011110000110000110000000000110011111111001100000000000001111000000000000000000000000000000011110000000110000000000000110000111111001100000000011 1g	1	70
JO22-S	JO-S	00000011000011110000000001100000011110000110000110011000000000001111111100000000000000000111100000000000000000000000000000000001100110000000000000110000111111000000000000000 0g	1	71
FQ10-M	FQ-M	00000011000011110000000111100000011110000111100110011000000000000111111000000000000000000111100000000000000000000000000000000000011000000000000000000110000000110000110011 1g	1	72
PN26-S	PN-S	00000011000011110000000111111000000111111110000110011000000000000111111000000110000000011110000000000000000000000000000000000000011000000000000000000110000111100001100111g	1	73
PN11-M	PN-M	00000011000011110000000111111000011110000110000111111000000000000011111111000011111111001111111100000000111100000000000000000000000000000000000000000001111000000000000110000 0g	1	74
FQ16-M	FQ-M	00000011000011110011111111100000011110000110000110000000001100000011001100000000000000000111100000000000000000000000011000000000011000000000000000000000000000000000001100111g	1	75
PN04-M	PN-M	00000011001100000000111111110000111100001100111100110000110011001111110000000000000000011110000000000000000000000000000000000000011000000000000000001111110000110000110000 1g	1	76
JO28-S	JO-S	00000011001111111000011001100000011110000110000110000000000000001111111100110000000000001111000000000000000000000000110011111111001100000000000000000011111100110000000001 11g	1	77
PN12-M	PN-M	00000011110000110011111111100000011110000110000110011000000000000000000000000001100000000111100000000000000000000000000000000000011000000000000000001111110000110000111111 1g	1	78
PN09-M	PN-M	00000011110011000011000011000000111100001100001111000000110000001111110000000000110000111100000000000000000000000000000000000000011000000000000000000111100001100111100001g	1	79
JO12-M	JO-M	00001100000000000000001111000000011110000110000110000000001100111111111100000000000000000111100000000000000000000111100000000000000000000000001100001111001100110000000001 11g	1	80
PN05-M	PN-M	00001111000000000000000111111000011110000110011110011000000000000000000000000001100000000111100000000000000000000000000000000000011000000000000000000111100001100001100001g	1	81
PN19-S	PN-S	00001111000000000000000111111110000111100111100001100110000110000111111110000001100000000111100000000000000000000000000000000000011000000000000000000111100001100001100111g	1	82

PN29-S	PN-S	0000111100001100000000001111000011110011110000110011000011000011111111000000000000000001111000000000000001100000000000000000000000011000000000000110000110000001100001100111g	1	83
JO07-M	JO-M	0000111100110000000011111100000011110000110000110011000000000011111111000000000000000001111000000000000001100001100000000000000110000000000000000011001100110000110000 1g	1	84
JO06-M	JO-M	0000111100111100000011111100000011110000110000110000000011000011111111000011001100000011110000000000001100000000000000000000001100111100000000000011000011110000111100 1g	1	85
PN03-M	PN-M	0000111111001100000011111111000011110000110011110000000011001111111111000000000000000001111000000000000000000000000000000000000011000000000000000111111000000000001100001g	1	86
JO13-M	JO-M	0011000000000000000000111100000011110000110000110000000011000011111111000011110000000011110000000000000000000011000000110011001100000000000110000111100000011000000000111g	1	87
JO30-S	JO-S	0011001100000011000000001100000011110000110000110000000000111111111111000000000000000001111000000001100000000001100000000110000110000000000110000001111110011000000000111g	1	88
JO05-M	JO-M	0011001100001100000000111100000011110000110000110000000000000011111111000011111100000011111111000011000000001100000011111111001100111100000000110011000000000000001100001g	1	89
JO14-M	JO-M	0011001100001100000011111100000011110000110000110000000000000011110011000011110000000011110000000000111100001111000000111100001100000000001100000011111100110000000000111g	1	90
JO26-S	JO-S	0011001100001100000011111111000011110000110000110000000000110011111111000000000000000001111000000000000000000000000000000000000011000000000000000000111111001100000000111g	1	91
JO27-S	JO-S	0011001100001100001100111100000011110000110000110000000000000011111111000000000000000001100000000000000000000000000000000000011110011000000000000000000111111001100000000111g	1	92
PN02-M	PN-M	0011001111001100111100001111000011110000110000110000000011000000111111000000110000110011110011000000110000000000000000000000000000000000000000000000111100000000110000001g	1	93
PN06-M	PN-M	0011001111001100111111111111000011110000110000110000000011000000110000000000000011000011110000000000000000000000000000000000000011000000110000000011111100000000000000001g	1	94
JO02-M	JO-M	1100001100111100000000111100000011110011000000110011000000000011111111000000110000000011110000000011111100000011000000000011001100000000000000000001100111111000011000001g	1	95
JO08-M	JO-M	1111001100000000000000110011000000111100001100001100000000000000111100110000000011000000111100000000000000000000000000000000000000110000000000001100001100111111000011110 01g	1	96

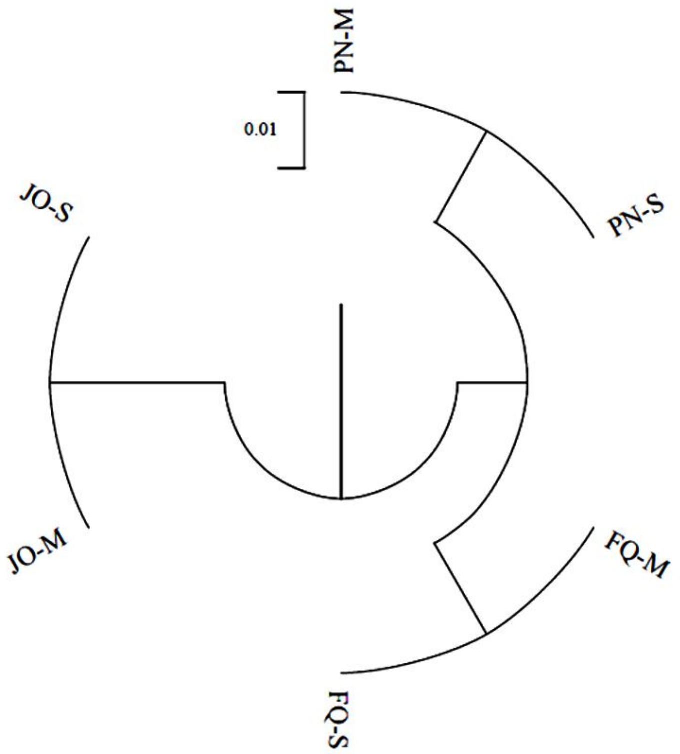

Supplementary Fig. S1. Phylogram showing Nei's genetic distances in *Bipolaris maydis* populations originating from single and multiple sweet corn cultivars at three sampling locations in Fujian Province. PN, FQ, and JO represent *B. maydis* isolates were collected from Pingnan, Fuqing, and Jian'ou regions in Fujian Province, respectively. S and M represent isolates were collected from the single and multiple corn cultivars, respectively. The phylogram was constructed on the basis of 13 inter-simple sequence repeat markers that clustered using the Neighbor-Joining method for multiple populations in MEGA 11 (Tamura et al. 2021).
